# Management and treatment of long COVID symptoms in general practices: An online-based survey

**DOI:** 10.3389/fpubh.2022.937100

**Published:** 2022-09-13

**Authors:** Anne Schrimpf, Annett Braesigk, Stefan Lippmann, Markus Bleckwenn

**Affiliations:** Department of General Practice, Faculty of Medicine, Leipzig University, Leipzig, Germany

**Keywords:** family practice, primary health care, post-acute COVID-19 syndrome, COVID-19, rehabilitation, outpatient care, investigational therapies

## Abstract

Independent from initial severity, many patients develop persistent symptoms after infection with SARS-CoV-2, described as long COVID syndrome. Most of these patients are treated by general practitioners (GPs). As evidence-based treatment recommendations are still sparse, GPs must make their therapy decisions under uncertainty. We investigated (1) the most frequently observed long COVID symptoms in general practices and (2) GPs' applied treatment and rehabilitation plans for these symptoms. In total, 143 German GPs participated in an online-based survey between 05/2021 and 07/2021. We found that each GP practice was treating on average 12 patients with long COVID symptoms. Most frequently seen symptoms were fatigue and reduced performance. Current therapy options were rated as poor and loss of smell and taste, fatigue, or lack of concentration were perceived to be especially difficult to treat. The use of drug and non-drug therapies and specialist referrals focused primarily on physiological and less on psychosomatic/psychological rehabilitation and followed guidelines of similar conditions. Our results provide first insights into how GPs approach a newly emerging condition in the absence of guidelines, evidence-based recommendations, or approved therapies, and might inform about GP preparedness in future pandemics. Our results also emphasize a gap between the current knowledge of the long COVID manifestation and knowledge about effective rehabilitation.

## Introduction

The coronavirus-19 disease (COVID-19) developed into a global pandemic and overwhelmed health care systems worldwide (1). Depending on comorbidities and the severity of COVID-19, individuals infected with the novel severe acute respiratory syndrome coronavirus (SARS-CoV-2) often exhibit symptoms such as fever, cough, fatigue, dyspnea, myalgia, and gastrointestinal symptoms in the acute phase of the infection (2–4). In addition, numerous hospitalized, but also non-hospitalized patients with acute COVID-19 have reported persistent symptoms lasting longer than 4 weeks after infection with the coronavirus (5, 6), and in some cases continuing longer than several months (7, 8). Symptoms lasting beyond the acute COVID-19 phase are described as long COVID syndrome (6, 9, 10), which is differentiated between symptoms that last between 4 and 12 weeks and symptoms that last more than 12 weeks (11).

The prevalence of long COVID is still under debate and varies between 10% and over 50% of patients diagnosed with COVID-19 (6, 10, 12–17). The most common long COVID symptoms are weakness, fatigue, concentration and memory impairments, and dyspnea, but also to a lesser extent headaches, muscle and joint pain, neuropathic pain, and decreased mental wellbeing (6, 12, 18–21). Cognitive symptoms might be attributable to alterations in the brain following an infection with SARS-CoV-2 (22), whereas other symptoms might be persistent due to elevated inflammatory cytokines and immunological activations (23). Rare cases of the development of autoimmune conditions, such as insulin-dependent diabetes mellitus, have also been reported (24).

The management of persistent symptoms after infection with SARS-CoV-2 is mainly provided ambulatory by general practitioners (GPs) (5, 21, 25). However, recommendations for ambulatory medical care of patients with long COVID are still not well-established and GPs primarily rely on comparisons with similar conditions (26). In addition, the large variety of symptoms associated with COVID-19 as well as their intermittent occurrence are increasing the difficulty to provide appropriate treatment and are requiring individualized rehabilitation plans (27). From patient reports at the beginning of the pandemic we know, that patients with persistent symptoms felt that their concerns were not taken seriously by their GPs (28, 29), which might be explained by the lack of evidence for effective management and treatment of single or multiple long COVID symptoms. Currently, several treatment and rehabilitation approaches have been proposed by national and international experts (20, 26, 30–34). A national long COVID guideline has been available since July 2021 in Germany and will be constantly adjusted based on emerging evidence (35). However, these guidelines and recommendations are not yet entirely backed up by scientific evidence and acknowledge the urgent need for further research on causal therapeutic approaches.

Considering the still emerging guidelines for medical practitioners (35) and the inconsistent information on the prevalence of long COVID in GP practices, we invited German GPs to participate in our online-based questionnaire study between May and July 2021. We aimed at assessing (1) the current number of patients with long COVID (patients with symptoms lasting between 4 and 12 weeks and lasting longer than 12 weeks) treated in general practices as well as the most frequently observed symptoms in patients with acute COVID-19 and long COVID seen in these practices. As a national guideline for long COVID was lacking at the time of the survey, we also investigated (2) GPs' applied treatment and rehabilitation plans for the most common long COVID symptoms and their decisions under uncertainty.

## Methods

### Recruitment procedure

**Figure 1:**
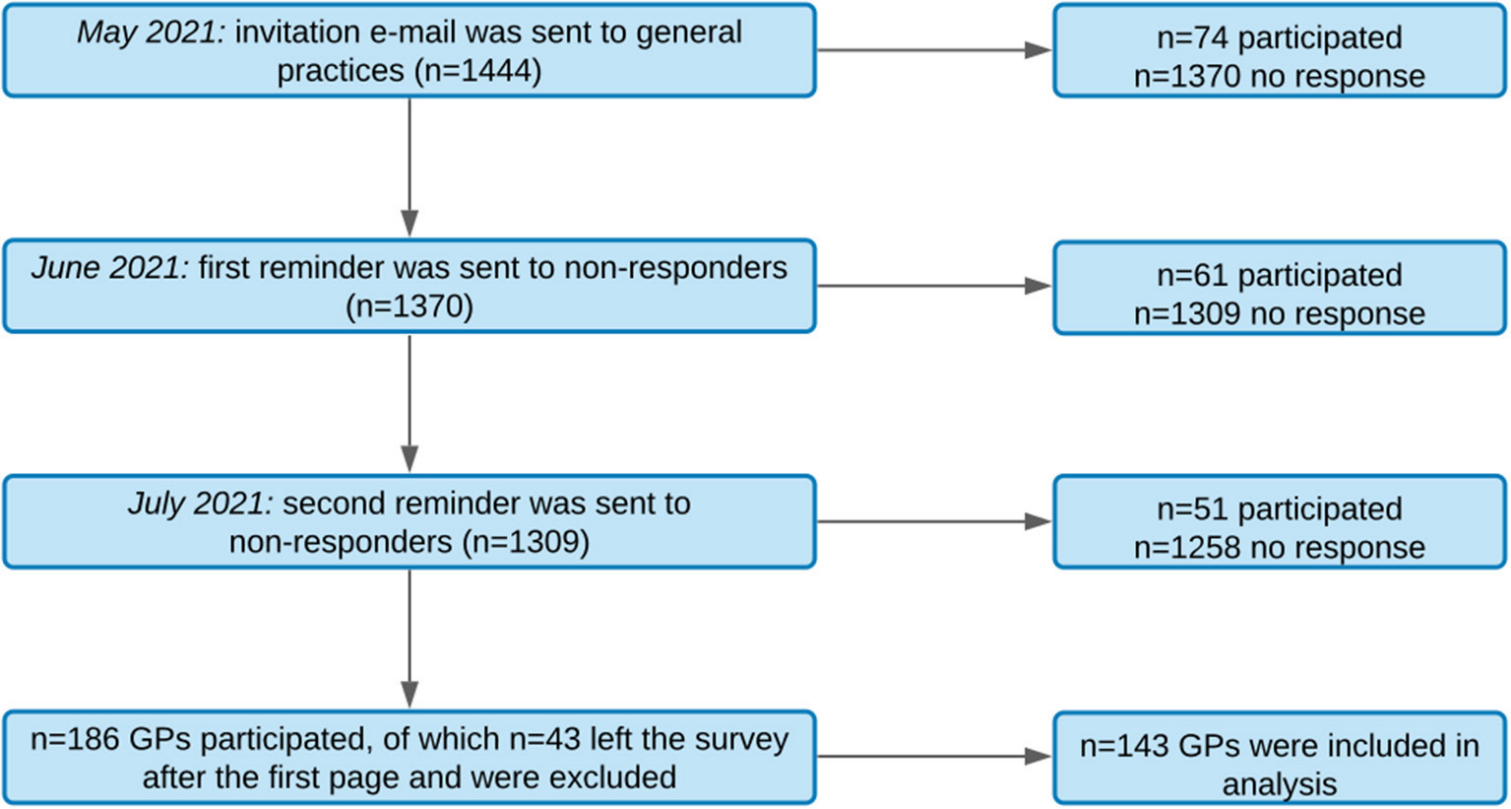
Flowchart of the recruitment process.

The data were collected in the Free State of Saxony, Germany, between May 2021 and July 2021. GPs were selected by availability of their e-mail addresses, which were obtained from the Association of Statutory Health Insurance Physicians Saxony (Kassenärztliche Vereinigung Sachsen). In 2020, a total of 1632 GPs with a mean age of 54 years were practicing in Saxony, of which 62.4% were female (36). GPs were invited by e-mail to voluntarily participate in this online survey. In May 2021, the first invitation e-mail was sent to 1444 GPs. The email contained information about the purpose of the survey, pseudonymous data handling, and a link to the online survey. The access to the survey was provided by an individual access code (token) to ensure that each GP participated only once. In June 2021, the first reminder containing the same information was sent to non-responders of the first e-mail [following the recommendations of (37)]. In July 2021, a second reminder was sent to non-responders of the second e-mail (see recruitment process in Figure 1).

### Survey

The questionnaire was self-developed in the Department of General Practice of the Leipzig University by an interdisciplinary research team (medical scientists, biochemist, and GPs) in a multi-stage revision process. The development process was complemented with extensive literature research aimed at identifying relevant factors for the treatment of long COVID. The final version can be found in Supplementary material S1. For the web-based evaluation, the software LimeSurvey (http://www.limesurvey.org/) was used, hosted on a secure server of the Leipzig University Computer Center. The completion of the online survey took ~15 min.

Participating GPs were asked to click the “I agree” button of the online informed consent form. Then, the survey started and comprised of the following topics: (1) demographics (e.g., age and sex), (2) current number of patients with acute and long COVID and their symptoms, (3) current treatment of long COVID symptoms. The response formats were choice answers, 4- and 5-point Likert scales, and free text entries. Likert scales for assessing the frequency of observed symptoms ranged from mostly, often, rarely, very rarely to never (5-points). Likert scales for assessing the current capabilities for diagnosis and therapy of acute COVID-19 and long COVID ranged from very good, rather good, rather poor to very poor (4-points).

Prior to implementation, the questionnaire underwent a think-aloud pre-testing (38) aimed at identifying problems or misunderstandings related to each item and to further develop the questionnaire. The provisional questionnaire was filled out by five GPs, who were instructed to think aloud while answering each item and report every spontaneous thought. After completing the questionnaire, the GPs were briefly interviewed about general issues with the questionnaire (e.g., length, structure, and general comprehensibility). After pre-testing, the provisional questionnaire was adjusted and further developed.

### Coding of free text entries

Participating GPs were asked to indicate the following, if needed, in free text fields: additional symptoms observed in acute and long COVID patients as well as medical and non-medical treatment and rehabilitation plans for 13 common long COVID symptoms. Free text entries were re-coded in major and subcategories by two authors of this study (AS, AB). Differences in coding were discussed until agreement was reached. Additional observed symptoms were in some cases aggregated if synonymous terms appeared in one field. Further, drugs were reported as generics, trade names, drug classes, or abbreviations. All drug entries were then grouped in the main category “drug class” and trade names or generics were coded as subcategories. As dosages or length of prescriptions were rarely stated, these statements were not analyzed.

### Ethics statement

The study was carried out in accordance with the Declaration of Helsinki and the study protocol was approved by the research ethics committee of the Leipzig University (reference number 157/21-ek). Online informed consent was obtained from all participants. They did not receive an incentive for their participation. No personal data besides age, sex, and education level were assessed.

### Statistical analyses

All statistical analyses were carried out using IBM SPSS Statistics 27 (Armonk, NY, USA). For descriptive statistics, missing values in single variables were considered by presenting frequencies as % (n/n_valid_). Continuous variables were presented as mean ± standard deviation (SD).

## Results

### Sample characteristics

Of the 186 GPs who participated in this study (13% total response rate), 43 GPs left the survey incomplete after the first page (demographic information). In total, 143 GPs were included in the analyses (mean age = 50.2 years, 61.1% female). GPs in our sample were slightly younger, but did not differ in gender distribution compared to the total population of GPs in Saxony.

### Acute COVID-19 and long COVID: Number of patients and observed symptoms in general practices

#### Patients with acute COVID-19

All GPs reported previously treating patients with acute COVID-19 in their practice. The current ability to diagnose acute COVID-19 in patients was rated as very good (76.9%) and current therapy options were rated as rather good (51.7%). For more details see Table 1.

**Table 1:** Number of GPs treating acute COVID-19 and long COVID, number of patients, and ratings of current diagnostic and therapeutic options in GP practices.

	**Acute COVID-19**	**Long COVID 4-12 weeks**	**Long COVID > 12 weeks**
Number of GPs treating this patient group	*n* = 143, 100%	*n* = 137, 97.2%	*n* = 113, 80.1%
Current number of patients per GP practice	n.a.	11.9	5.9
Current ability to diagnose		
Very good	76.9%	19.7%	19.5%
Rather good	18.2%	62.8%	40.7%
Rather poor	1.4%	16.8%	35.4%
Very poor	0%	0%	3.5%
No answer	3.5%	0.7%	0.9%
Current therapy options			
Very good	29.4%	6.6%	4.4%
Rather good	51.7%	41.7%	21.2%
Rather poor	12.6%	47.4%	54.9%
Very poor	2.1%	3.6%	18.6%
No answer	4.2%	0.7%	0.9%

Data are presented as percentage (n/n_valid_). Current diagnostic and therapeutic options for acute COVID-19 and long COVID were assessed by using 4-point rating scales, each ranging from very good to very poor. N.a., not assessed.

**Figure 2:**
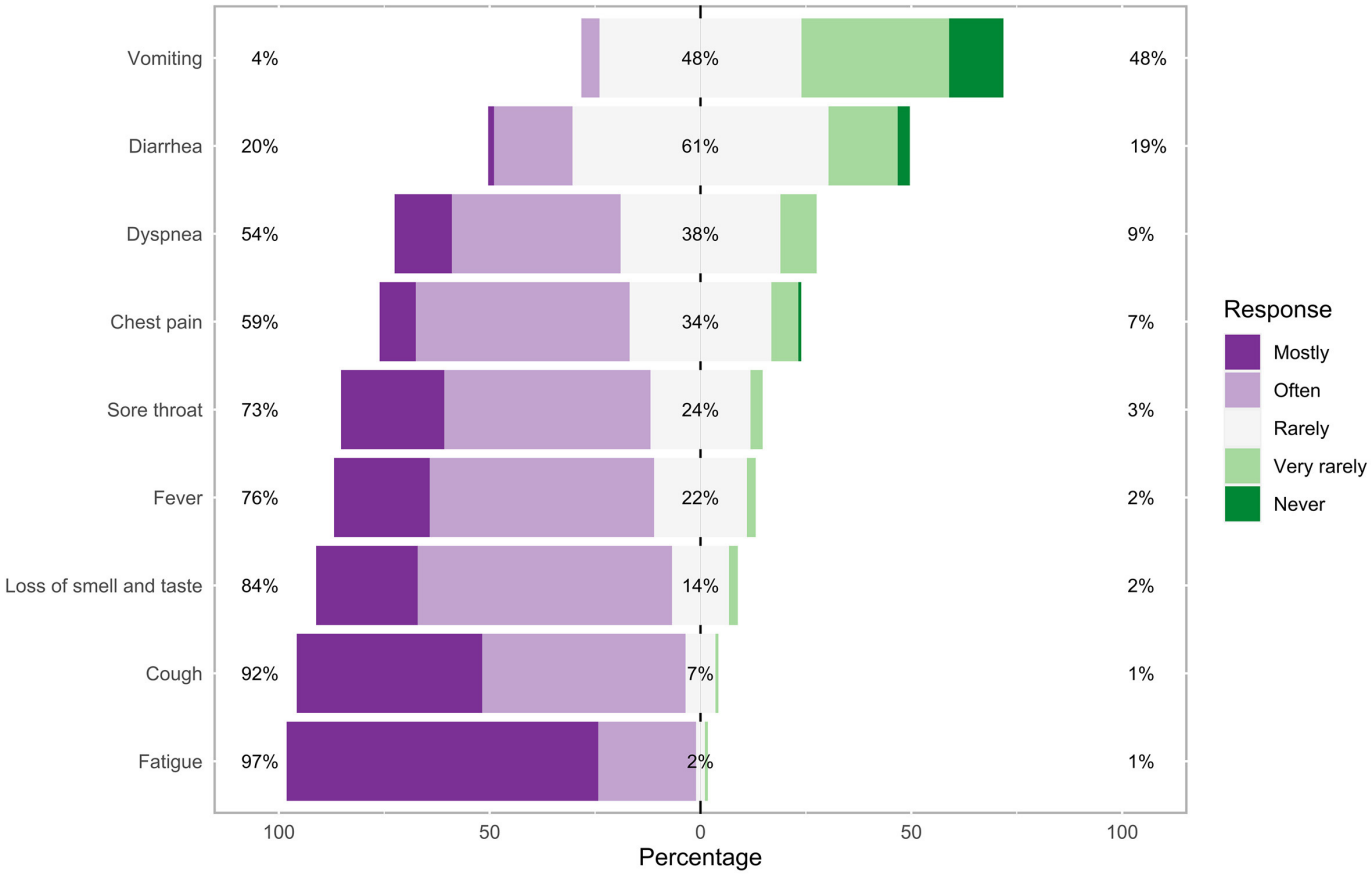
Frequencies of each observed symptom in patients with acute COVID-19 in the general practice. Percentages on the left and right side of the graph represent a summary of mostly and often on the left and very rarely and never on the right side.

The COVID-19 symptoms most frequently observed by participating GPs were fatigue (73.9%) and cough (44%). The frequencies of each observed symptom can be found in Figure 2. GPs were asked to report additional symptoms they observed in their patients with an acute COVID-19 infection that were not already listed in the questionnaire. Results of these reports can be found in Table 2.

**Table 2:** Additional symptoms of patients with acute COVID-19 reported by GPs.

**Major category**	**Subcategory**	n*	%**
Pain symptoms		90	62.9
	Headaches	36	25.2
	Limb pain	30	21.0
	Joint pain	8	5.6
	Muscle pain	7	4.2
	Back pain	4	2.8
	Stomachaches	3	2.1
	General pain	1	0.7
	Earaches	1	0.7
	Retrobulbar pain	1	0.7
Psychological symptoms and behavioral disorders		16	11.2
	Concentration disorders	6	4.2
	Memory impairments	5	3.5
	Anxiety	3	2.1
	Depressive symptoms	1	0.7
	Apathy	1	0.7
Perceptual disorders		13	9.1
	Vertigo	10	7.0
	Restlessness	2	1.4
	Dizziness	1	0.7
General condition		13	9.1
	Weakness/exhaustion	6	4.2
	Loss of appetite	2	1.4
	Chills	2	1.4
	Night sweat	1	0.7
	Weight loss	1	0.7
	Edemas	1	0.7
Symptoms of the circulatory and respiratory system		8	5.6
	Blood pressure fluctuations	3	2.1
	Cardiac arrhythmia	2	1.4
	Tightness in the chest	1	0.7
	Irregular heart rhythm	1	0.7
	Acrocyanosis	1	0.7
Symptoms of the skin		6	4.2
	Exanthema	4	2.8
	Hair loss	1	0.7
	Paresthesia	1	0.7
Symptoms of eyes and nose		6	4.2
	Rhinitis	3	2.1
	Pressure in paranasal sinus	1	0.7
	Vision loss	1	0.7
	Burning eyes	1	0.7
Symptoms of nervous system		5	3.5
	Insomnia	2	1.4
	Seizures	1	0.7
	Transient ischemic attack	1	0.7
	Muscle weakness	1	0.7
Symptoms of other organs or abnormal values		5	3.5
	Urinary tract infection	2	1.4
	Stomach problems	1	0.7
	Drop in oxygen partial pressure	1	0.7
	Nausea	1	0.7

^*^ = statements in this category,

^**^ = % of GPs who stated an insight from this category.

#### Patients with long COVID symptoms lasting between 4 and 12 weeks

Most GPs (97.2%) reported previously treating patients with long COVID symptoms lasting between 4 and 12 weeks in their practice. On average, 11.9 of these patients were currently treated in each practice. The current ability to diagnose long COVID (4–12 weeks) in patients was mainly rated as rather good (62.8%), whereas current therapy options were rated as rather poor (47.4%). For more details see Table 1.

**Figure 3:**
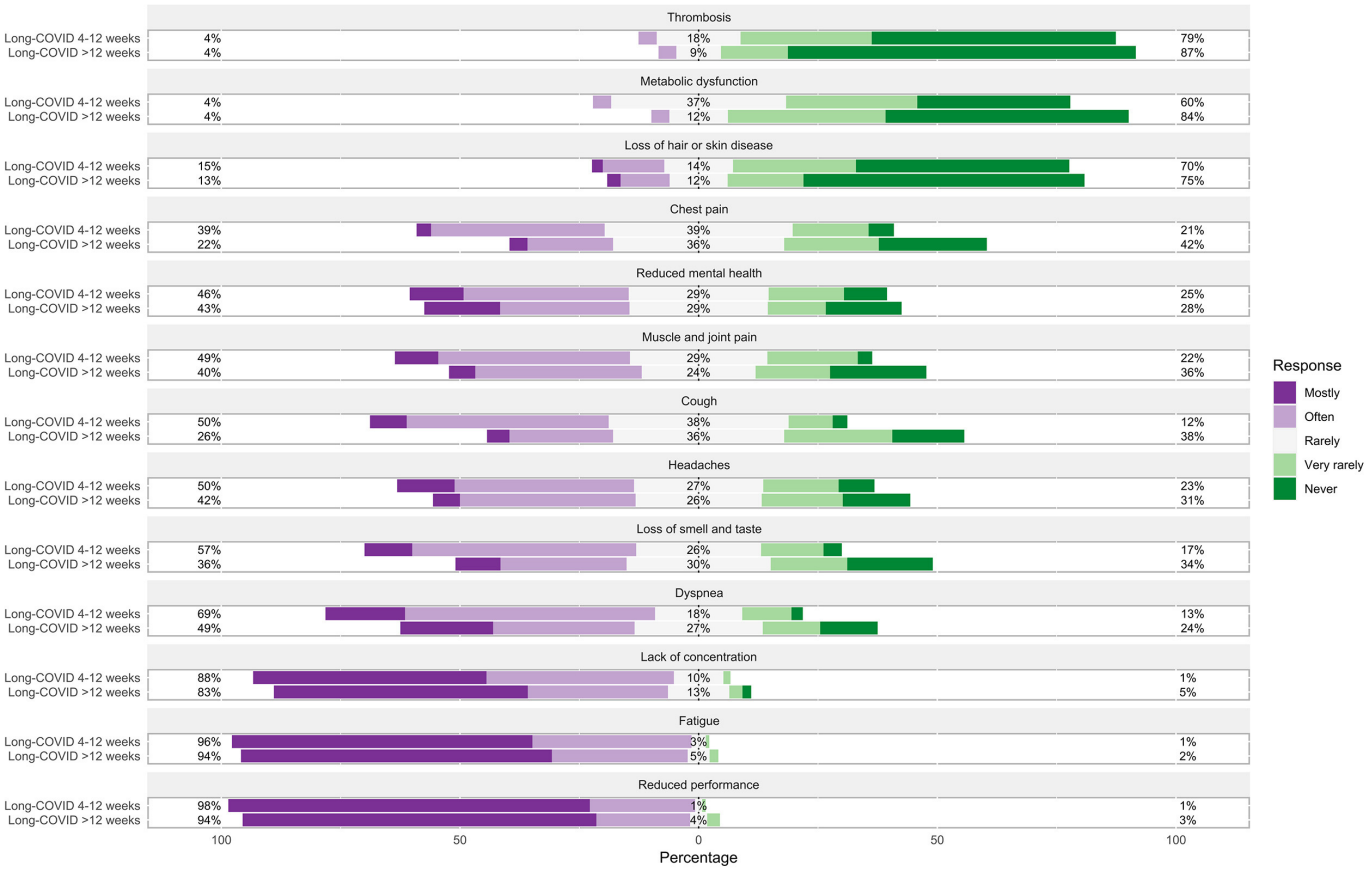
Frequencies of each observed symptom in patients with long COVID symptoms lasting between 4 and 12 weeks and lasting more than 12 weeks in the general practice. Percentages on the left and right side of the graph represent a summary of mostly and often on the left and very rarely and never on the right side.

Long COVID symptoms lasting between 4 and 12 weeks that were most frequently observed by participating GPs were fatigue (63%) and reduced performance (75.7%). The frequencies of each observed symptom can be found in Figure 3. GPs were asked to report additional symptoms they observed in their patients with long COVID symptoms lasting between 4 and 12 weeks that were not already listed in the questionnaire. Results of these reports can be found in Table 3.

**Table 3:** Additional reported symptoms of patients with long COVID lasting between 4 and 12 weeks.

**Major category**	**Subcategory**	n*	%**
Symptoms of the circulatory and respiratory system		10	7.3
	Cardiac arrhythmia	4	2.9
	Hypertension	2	1.5
	Blood pressure fluctuations	1	0.7
	Tightness in the chest	1	0.7
	Irregular heart rhythm	1	0.7
	Abnormal sensations in respiratory tract	1	0.7
Psychological symptoms and behavioral disorders		8	5.8
	Memory impairments	2	1.5
	Anxiety	2	1.5
	Depressive symptoms	1	0.7
	Somatization	1	0.7
	Psychological stress	1	0.7
	Instability	1	0.7
General condition		7	5.1
	Exacerbation of pre-existing conditions	3	2.2
	Weakness/exhaustion	2	1.5
	Loss of appetite	1	0.7
	Perspiration	1	0.7
Symptoms of nervous system		6	4.4
	Insomnia	2	1.5
	Neuropathic symptoms	2	1.5
	Abnormal sensations in muscles	1	0.7
	Exacerbation of multiple sclerosis	1	0.7
Symptoms of other organs or abnormal values		5	3.6
	Exacerbation of rheumatism	2	1.5
	Dysbiosis	1	0.7
	Elevated erythrocyte sedimentation rate	1	0.7
	Irritable bowel syndrome	1	0.7
Perceptual disorders			
	Vertigo	3	2.2
Symptoms of the skin		2	1.5
	Skin alterations	1	0.7
	Paresthesia	1	0.7
Symptoms of ears and nose		2	1.5
	Sinusitis	1	0.7
	Tinnitus	1	0.7

^*^ = statements in this category,

^**^ = % of GPs who stated an insight from this category and already treated this patient group (n = 137).

#### Patients with long COVID symptoms lasting more than 12 weeks

In this sample, 79.6% of GPs reported previously treating patients with long COVID symptoms lasting more than 12 weeks in their practice. On average, 5.9 of these patients were currently treated in each practice. The current ability to diagnose long COVID (>12 weeks) in patients was most often rated as rather good (40.7%), whereas current therapy options were rated as rather poor (54.9%). For more details see Table 1.

Long COVID symptoms lasting more than 12 weeks that were most frequently observed by participating GPs were fatigue (65.1%) and reduced performance (74.1%). The frequencies of each observed symptom can be found in Figure 3. GPs were asked to report additional symptoms they observed in their patients with long COVID symptoms lasting more than 12 weeks that were not already listed in the questionnaire. Results of these reports can be found in Table 4.

**Table 4:** Additional reported symptoms of patients with long COVID lasting more than 12 weeks.

**Major category**	**Subcategory**	n*	%**
Psychological symptoms and behavioral disorders		9	8.0
	Depressive symptoms	3	2.7
	Hypersensitivity	3	2.7
	Memory impairments	1	0.9
	Somatization	1	0.9
	Anxiety	1	0.9
Symptoms of nervous system		6	5.3
	Insomnia	4	3.5
	Neuropathic symptoms	2	1.8
Symptoms of the circulatory and respiratory system		4	3.5
	Hypertension	2	1.8
	Exercise-induced dyspnea	2	1.8
General condition		4	3.5
	Weakness/exhaustion	2	1.8
	Exacerbation of pre-existing conditions	1	0.9
	Weight loss	1	0.9
Symptoms of other organs or abnormal values		3	2.7
	Renal insufficiency	1	0.9
	Dysbiosis	1	0.9
	Blood sugar imbalances	1	0.9
Perceptual disorders			
	Vertigo	1	0.9
Symptoms of the skin			
	Paresthesia	1	0.9
Symptoms of eyes			
	Vision loss	1	0.9

^*^ = statements in this category,

^**^ = % of GPs who stated an insight from this category and already treated this patient group (n = 113).

### Treatment and rehabilitation of patients with acute COVID-19 and long COVID

#### Rehabilitation of patients with acute COVID-19 and long COVID

Participating GPs estimated that on average 18.3% of all patients with COVID-19 are currently in need of a certificate of incapacity for work longer than 6 weeks. Further, they reported that ~3.7% of patients with COVID-19 currently have access to rehabilitation centers.

#### Treatment of long COVID symptoms

**Figure 4:**
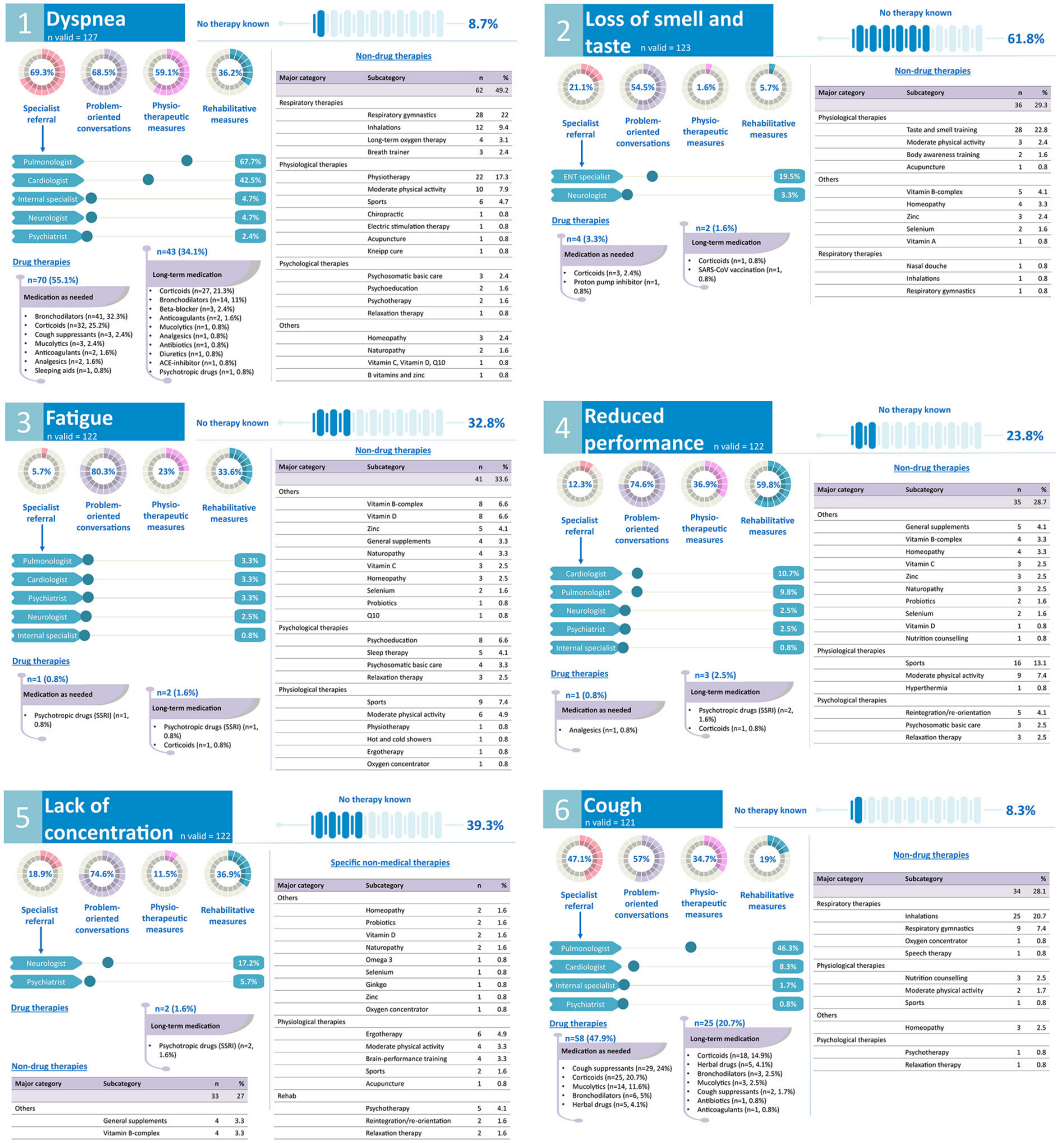
GPs' treatment and rehabilitation plans of common long COVID symptoms: dyspnea, loss of smell and taste, fatigue, reduced performance, lack of concentration, cough. Kneipp cure, hydrotherapy; Q10, coenzyme Q10.

**Figure 5:**
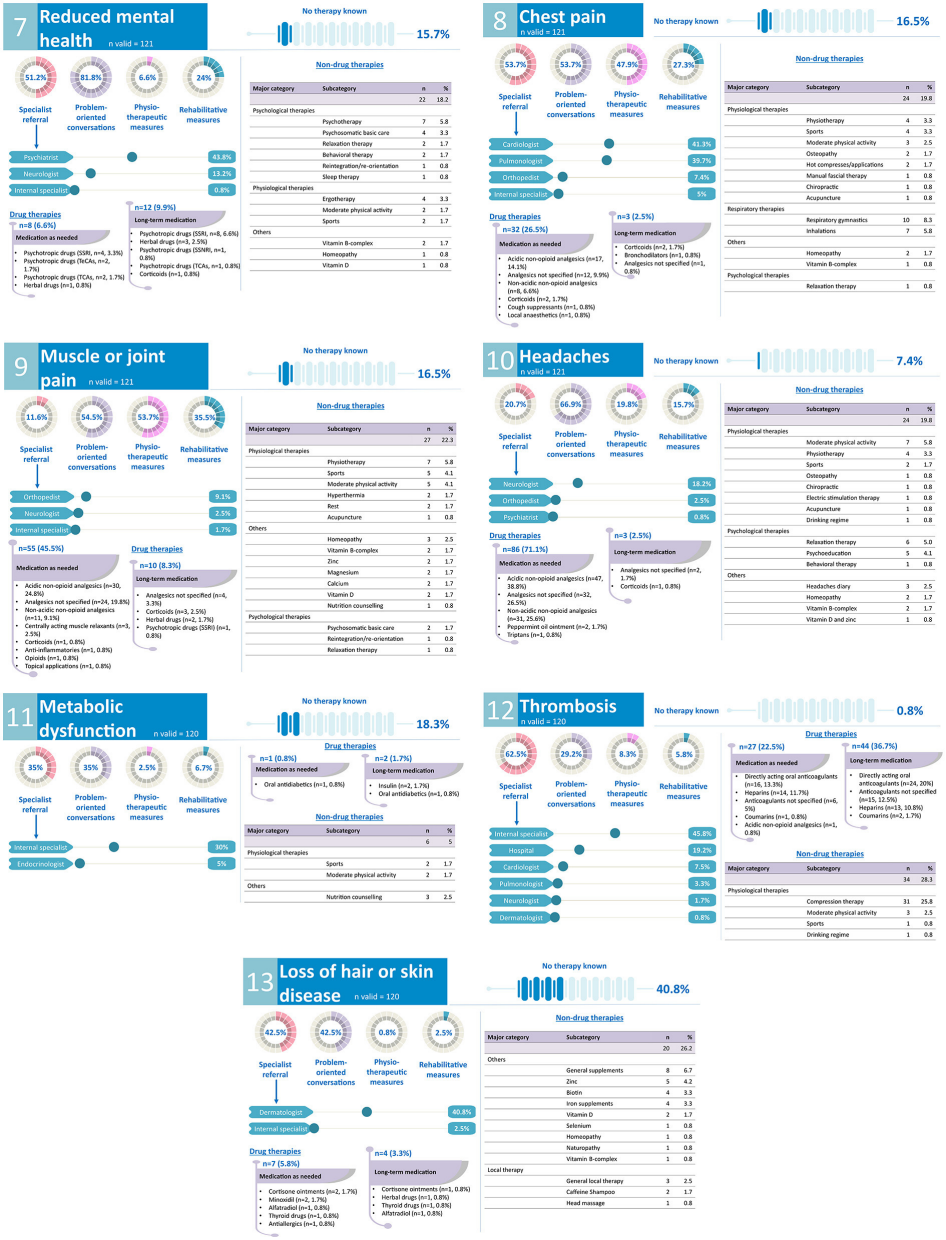
GPs' treatment and rehabilitation plans of common long COVID symptoms: reduced mental health, chest pain, muscle or joint pain, headaches, metabolic dysfunction, thrombosis, loss of hair or skin disease. SSNRI, selective serotonin noradrenalin reuptake inhibitor; SSRI, selective serotonin reuptake inhibitor; TeCAs, tetracyclic antidepressants; TCAs, tricyclic antidepressants.

Participating GPs were asked to give information about their usual recommended therapy for 13 common long COVID symptoms. For each symptom, GPs should indicate the following: if they know of an appropriate therapy, suitable medications (short- and long-term administration), non-drug therapies (e.g., physiotherapy, rehabilitation, etc.), and/or referrals to specialists. A summary of the findings can be found in the infographic (Figures 4, 5).

## Discussion

The present study investigated the number of patients with long COVID, currently observed long COVID symptoms, as well as their treatment in 143 German general practices. Fatigue and reduced performance were the most frequent symptoms observed by GPs in patients with long COVID. At the same time, treatment options for common long COVID symptoms were rated as poor and loss of smell and taste, fatigue, or lack of concentration were perceived to be especially difficult to treat. Drug and non-drug therapies prescribed by GPs primarily focused on physiological and less on psychological rehabilitation of patients with long COVID. Our results provide first insights into how GPs approach a newly emerging condition under uncertainty and without direct curative approaches. The results might also provide additional guidance for GPs worldwide.

### Number of patients with long COVID and observed symptoms

First, we assessed the current number of patients with long COVID treated in GP practices. Almost all participating GPs had previously treated patients with persistent symptoms after SARS-CoV-2 infection. On average, each GP practice was treating 12 patients with symptoms lasting between 4 and 12 weeks after infection with the coronavirus between May and July 2021. During this survey period, approximately six patients with long COVID symptoms lasting longer than 12 weeks were treated by each participating GP. Earlier in the pandemic, 53.8% of French GPs treated at least one patient with long COVID in their practices, particularly in the most strongly affected areas (39). With the progression of the pandemic, it is conceivable that the long COVID syndrome will play a lasting role in GP practices.

We also assessed the most frequent symptoms in patients with acute COVID-19 and long COVID who presented in participating practices. In line with previous research (2–4), GPs most frequently observed cough, fatigue, fever, and loss of smell and taste in patients with acute COVID-19, indicating that the patient population seeking treatment by their GP and with a potentially milder courses of the disease exhibited a comparable spectrum of symptoms than, for example, clinical patient populations. Free text analyses revealed recurring observations of pain symptoms, especially aching head and limbs. Around one in five of the patients with COVID-19 presenting in GP practices in our study were on sick leave for more than 6 weeks due to the severity of their symptoms, which is consistent with previous studies (40).

In patients who reported symptoms lasting between 4 and 12 weeks, GPs frequently observed cognitive and psychosomatic dysfunctions, such as fatigue, reduced performance, and lack of concentration, persistent respiratory dysfunctions, such as cough and dyspnea, as well as loss of smell and taste, muscle and joint pain, and headaches. Our results are in line with patient reports on the most pervasive symptoms (3, 6, 7, 14, 21, 41–43). Additionally, GPs mainly observed cognitive and psychosomatic dysfunctions, such as fatigue, reduced performance, and lack of concentration, in patients with persistent symptoms lasting longer than 12 weeks, which is in accordance with previous reports (6, 42). Although depressive symptoms or anxiety have been found to be common in patients with long COVID (44, 45), participating GPs rarely mentioned these symptoms in our study. Potential explanations could be that GPs treat to a great extent patients with a milder course of the disease and therefore with a lower prevalence of depressive symptoms. However, while some studies found fewer depressive symptoms and anxiety in non-hospitalized compared to hospitalized patients (14) and in ward patients compared to ICU patients (46), other studies found no relationship between initial disease severity and depression (45, 47), indicating that further research is urgently needed. It is further conceivable, that the frequent occurrence of fatigue and lack of concentration reported by GPs is associated with underlying depressive symptoms, as has been shown in previous studies (48, 49). Finally, the diagnosis of depression, especially in less severe cases, has been found to be low among GPs (50–52). Besides a lack of knowledge, several reasons have been discussed: GPs might want to exclude somatic diseases first and observe the symptoms and the circumstances over a longer period of time before diagnosing depression and initiating a respective treatment (50, 53). Further, patients might underreport mental symptoms due to associated stigma (50, 53, 54). Lastly, patients without prior depressive episodes have been found to be less likely diagnosed as depressed by their GPs (50, 55), indicating that newly emerging depressive symptoms following infection with SARS-CoV-2 might be less likely recognized.

Our results suggest that the long COVID syndrome is widely established in German GP practices and that addressing cognitive, psychosomatic, and respiratory impairments plays an especially prominent role in the treatment of these patients.

### Treatment of and rehabilitation plans for common long COVID symptoms

In addition, we investigated GPs' strategies to treat patients with long COVID symptoms. At the time of the survey, a national guideline for long COVID and causal therapies were lacking, suggesting that GPs made their treatment decisions under uncertainty (56–58) and may have focused on symptom-oriented treatment. In the meantime, several management recommendations for the outpatient setting have been proposed (26, 31, 34, 59–61) and a national guideline was established. In our study, most GPs rated the current treatment options for long COVID as poor or rather poor. Symptoms such as loss of smell and taste, fatigue, lack of concentration, loss of hair, or skin diseases might be particularly difficult to manage for some GPs, indicating that treatment options for these symptoms are limited or symptoms are difficult to define. These perceptions are in line with a recent review showing a discrepancy in the literature between the current knowledge about long COVID characteristics and rehabilitation recommendations (60). In contrast, GPs felt more confident in treating symptoms such as thrombosis, cough, headaches, or dyspnea, which might be due to more available medication, treatment, or referral options.

Further, our results show the use of a variety of medications for the treatment of long COVID symptoms. Compared to previous studies reporting an increased prescription of triptans in case of symptoms such as headaches or migraine (5), GPs in our study rarely utilized triptans and instead mainly prescribed acidic and non-acidic non-opioid analgesics (e.g., paracetamol, ibuprofen) to patients with long COVID presenting with headaches. We also did not find an excessive prescription of antibiotics, which has been reported elsewhere (62). However, the use of corticoids or bronchodilators for respiratory symptoms were common, which has also been found in other studies (5, 62). Notably, none of the drugs have been approved for the treatment of long COVID so far and their safety and effectiveness needs to be further evaluated (63). Further, some GPs also recommended a variety of supplements, such as vitamin D and vitamin B-complex. Whereas, a higher vitamin D level might be protective against a worse outcome after infection with SARS-CoV-2 (64), the therapeutic effects of vitamin D supplementation have not yet been confirmed (65). To our knowledge, the evidence for therapeutic effects of B vitamins is to date non-existent.

In addition to drug therapies, GPs in our study often referred patients to physical therapies and moderate exercises to improve symptoms and performance, which have also been recommended by current research (32, 60, 66). However, physical exercises can also trigger symptom relapses, especially in patients experiencing dyspnea, muscle and joint pain, or chest pain (7, 34). In addition, reports from patients with long COVID highlight the need for an individually tailored physical activity plan establishing thresholds to avoid relapses (28, 67). Particularly, unadjusted physical activity plans and a subsequent worsening of symptoms might discourage patients from further training (67). Further, in accordance with a recent review (60), the reported use of non-drug therapies for patients with long COVID in our study focused primarily on exercise and physical rehabilitation, whereas the use of educational, behavioral, or psychological therapies was low. Given the potential complex sequalae following an infection with SARS-CoV-2, a more comprehensive and holistic rehabilitation is recommended.

Our results also show that specialist referrals for long COVID symptoms were common, which might increase coordination difficulties for GPs, especially in patients with more complex symptoms. The urgent need for multi-disciplinary rehabilitation teams addressing persistent symptoms and a low threshold for referrals has been discussed previously (26, 32, 60), however, access to specialists as well as their perceived insufficient communication with GPs might hamper effective therapy of patients experiencing long COVID symptoms (29, 56).

### Limitations

The treatment of patients with long COVID was assessed *via* individual symptoms rather than by often occurring groups of symptoms, potentially leaving some more holistic treatment strategies unreported. Further, as our data were collected in mid-2021, our results might not be applicable to long COVID manifestations caused by other SARS-CoV-2 variants occurring after the study period (e.g., Omicron). We also did not assess potential differences in long COVID symptoms in hospitalized and non-hospitalized patients in the acute phase of the SARS-CoV-2 infection. Another limitation is the absence of patient data or case reports for each therapy and treatment strategy reported by GPs in our study. This also includes potentially differing treatment strategies applied by GPs in patients with pre-existing illnesses or co-morbidities to avoid, for instance, drug-drug-interactions (68) or a worsening of the underlying disease (69). Further, we did not ask GPs about additional diagnostic methods, such as X-rays or blood tests, potentially used to guide their decision. Given the nature of our study, a selection bias might have occurred. Lastly, we did not conduct a power analysis, wherefore the data are to be considered exploratory and cannot be generalized.

### Implications

Our study contributes information on the current state of treatment and rehabilitation plans for patients with long COVID symptoms presenting in German GP practices. As evidence-based management recommendations and approved drugs were absent at the time of the survey, our results indicate that GPs relied on their experience with related conditions when treating patients with persistent symptoms after infection with SARS-CoV-2. Further, as research is constantly advancing the knowledge about long COVID management, GPs should be informed regularly about new or adjusted options and newly emerging treatment programs, e.g., through workshops. However, our results also emphasize a gap between the current knowledge of the long COVID manifestation and knowledge about effective rehabilitation. The current German national guideline on post-/long COVID published shortly after our survey was conducted provides broad recommendations but lacks specific evidence-based guidance on the treatment of some symptoms. Research and health authorities should especially focus on the development of holistic and multi-professional treatment plans for common, but more ambiguous long COVID symptoms, that are still difficult to manage for GPs, such as loss of smell and taste, fatigue, or lack of concentration.

## Conclusion

GPs are on the front line managing patients with long COVID. We confirm that especially ambiguous long COVID symptoms might be challenging to treat, and that GPs primarily rely on their experience with similar conditions. Further, psychosomatic or psychological rehabilitation of patients with long COVID was underrepresented and might need further attention. Our findings provide a rare insight into how GPs manage a new condition in the absence of guidelines, evidence-based recommendations, or approved therapies and how GPs develop their treatment strategies. The collective therapy strategies presented in this study might also inform other GPs worldwide to guide their treatment decisions.

## Data availability statement

The raw data supporting the conclusions of this article will be made available by the authors, without undue reservation.

## Ethics statement

The studies involving human participants were reviewed and approved by Research Ethics Committee of the Leipzig University. The patients/participants provided their written informed consent to participate in this study.

## Author contributions

AS, AB, SL, and MB designed the study. AS and AB prepared the study, collected, and analyzed the data. AS wrote the manuscript. AB, SL, and MB reviewed and edited the manuscript. All authors contributed to the article and approved the submitted version.

## Conflict of interest

The authors declare that the research was conducted in the absence of any commercial or financial relationships that could be construed as a potential conflict of interest.

## Publisher's note

All claims expressed in this article are solely those of the authors and do not necessarily represent those of their affiliated organizations, or those of the publisher, the editors and the reviewers. Any product that may be evaluated in this article, or claim that may be made by its manufacturer, is not guaranteed or endorsed by the publisher.
