# Supplementary material for: Management and treatment of long COVID symptoms in general practices: An online-based survey
Source: Front Public Health. 2022 Sep 13;10:937100. doi: 10.3389/fpubh.2022.937100 (PMC9513068; doi:10.3389/fpubh.2022.937100)
Supplement: Supplementary file 1 [file Table_1.docx]

**Supplements**

**Supplementary material S1.** GP questionnaire translated into English.

| 1. Demographics | | | | | |
| --- | --- | --- | --- | --- | --- |
| Age: | __ __ years | Sex: | □ female | □ male | □ diverse |

| 2. Have you ever treated patients with acute COVID infection in your practice? | | | | | | |
| --- | --- | --- | --- | --- | --- | --- |
| □ **Yes** | | | □ **No** | | | |
| **If yes**, what symptoms do you observe in your patients acutely infected with COVID? | | | | | | |
| *Please indicate frequency.* | **Mostly** | **Often** | | **Rarely** | **Very rarely** | **Never** |
| Dyspnea | □ | □ | | □ | □ | □ |
| Fever | □ | □ | | □ | □ | □ |
| Loss of smell and taste | □ | □ | | □ | □ | □ |
| Diarrhea | □ | □ | | □ | □ | □ |
| Vomiting | □ | □ | | □ | □ | □ |
| Fatigue | □ | □ | | □ | □ | □ |
| Cough | □ | □ | | □ | □ | □ |
| Sore throat | □ | □ | | □ | □ | □ |
| Chest pain | □ | □ | | □ | □ | □ |
| Other symptoms: |  | | | | | |

| 3. Have you ever treated patients with long COVID syndrome (4-12 weeks after diagnosis) in your practice? | | | | | | | |
| --- | --- | --- | --- | --- | --- | --- | --- |
| □ **Yes** | | | □ **No** | | | | |
| **If yes,** how many patients with long COVID syndrome (4-12 weeks after diagnosis) do you approximately currently treat in your practice (total number)? | | | | | | __ __ | |
| **If yes**, what symptoms do you observe in your patients with long COVID syndrome (4-12 weeks after diagnosis)? | | | | | | | |
| *Please indicate frequency.* | **Mostly** | **Often** | | **Rarely** | **Very rarely** | | **Never** |
| Dyspnea | □ | □ | | □ | □ | | □ |
| Loss of smell and taste | □ | □ | | □ | □ | | □ |
| Fatigue | □ | □ | | □ | □ | | □ |
| Reduced performance | □ | □ | | □ | □ | | □ |
| Lack of concentration | □ | □ | | □ | □ | | □ |
| Cough | □ | □ | | □ | □ | | □ |
| Reduced mental health | □ | □ | | □ | □ | | □ |
| Chest pain | □ | □ | | □ | □ | | □ |
| Muscle or joint pain | □ | □ | | □ | □ | | □ |
| Headaches | □ | □ | | □ | □ | | □ |
| Metabolic dysfunction | □ | □ | | □ | □ | | □ |
| Thrombosis | □ | □ | | □ | □ | | □ |
| Loss of hair or skin disease | □ | □ | | □ | □ | | □ |
| Other symptoms: |  | | | | | | |

| 4. Have you ever treated patients with long COVID syndrome (>12 weeks after diagnosis) in your practice? | | | | | | | |
| --- | --- | --- | --- | --- | --- | --- | --- |
| □ **Yes** | | | □ **No** | | | | |
| **If yes,** how many patients with long COVID syndrome (>12 weeks after diagnosis) do you approximately currently treat in your practice (total number)? | | | | | | __ __ | |
| **If yes**, what symptoms do you observe in your patients with long COVID syndrome (>12 weeks after diagnosis)? | | | | | | | |
| *Please indicate frequency.* | **Mostly** | **Often** | | **Rarely** | **Very rarely** | | **Never** |
| Dyspnea | □ | □ | | □ | □ | | □ |
| Loss of smell and taste | □ | □ | | □ | □ | | □ |
| Fatigue | □ | □ | | □ | □ | | □ |
| Reduced performance | □ | □ | | □ | □ | | □ |
| Lack of concentration | □ | □ | | □ | □ | | □ |
| Cough | □ | □ | | □ | □ | | □ |
| Reduced mental health | □ | □ | | □ | □ | | □ |
| Chest pain | □ | □ | | □ | □ | | □ |
| Muscle or joint pain | □ | □ | | □ | □ | | □ |
| Headaches | □ | □ | | □ | □ | | □ |
| Metabolic dysfunction | □ | □ | | □ | □ | | □ |
| Thrombosis | □ | □ | | □ | □ | | □ |
| Loss of hair or skin disease | □ | □ | | □ | □ | | □ |
| Other symptoms: |  | | | | | | |

| 5. How do you currently assess the capabilities for diagnosis and therapy of the following patient groups in your practice? | | | | |
| --- | --- | --- | --- | --- |
|  | **Very good** | **Rather good** | **Rather poor** | **Very poor** |
| **Diagnosis of:** | □ | □ | □ | □ |
| Acute COVID | □ | □ | □ | □ |
| Long COVID (4-12 weeks after diagnosis) | □ | □ | □ | □ |
| Long COVID (> 12 weeks after diagnosis) | □ | □ | □ | □ |
| **Therapy of:** | □ | □ | □ | □ |
| Patients with acute COVID | □ | □ | □ | □ |
| Patients with long COVID (4-12 weeks after diagnosis) | □ | □ | □ | □ |
| Patients with long COVID (> 12 weeks after diagnosis) | □ | □ | □ | □ |

| 6. Rehabilitation | |
| --- | --- |
| How many of your patients with COVID-19 do you estimate need a certificate of incapacity for more than six weeks? | __ __ __ % |
| How many of your patients with COVID-19 do you estimate have access to rehabilitation centers? | __ __ __ % |

| 7. In the following, there will be a number of possible Long-COVID symptoms listed. We are interested in how you currently treat these symptoms. If the symptoms have not yet appeared in your patients, we are interested in how you would treat them. | |
| --- | --- |
| *Multiple responses possible.* | **Therapy** |
| Dyspnea | □ Drug therapies  □ Medication as needed:________  □ Long-term medication:________  □ Rehabilitative measures  □ Physiotherapeutic measures  □ Problem-oriented conversations  □ Specialist referral  □ Internal specialist □ Pulmonologist □ Cardiologist  □ Orthopedist □ Neurologist □ Psychiatrist  □ Dermatologist □ Others: _____________________  □ Other non-drug therapies: _________  □ No therapy known |
| Loss of smell and taste | □ Drug therapies  □ Medication as needed:________  □ Long-term medication:________  □ Rehabilitative measures  □ Physiotherapeutic measures  □ Problem-oriented conversations  □ Specialist referral  □ Internal specialist □ Pulmonologist □ Cardiologist  □ Orthopedist □ Neurologist □ Psychiatrist  □ Dermatologist □ Others: _____________________  □ Other non-drug therapies: _________  □ No therapy known |
| Fatigue | □ Drug therapies  □ Medication as needed:________  □ Long-term medication:________  □ Rehabilitative measures  □ Physiotherapeutic measures  □ Problem-oriented conversations  □ Specialist referral  □ Internal specialist □ Pulmonologist □ Cardiologist  □ Orthopedist □ Neurologist □ Psychiatrist  □ Dermatologist □ Others: _____________________  □ Other non-drug therapies: _________  □ No therapy known |
| Reduced performance | □ Drug therapies  □ Medication as needed:________  □ Long-term medication:________  □ Rehabilitative measures  □ Physiotherapeutic measures  □ Problem-oriented conversations  □ Specialist referral  □ Internal specialist □ Pulmonologist □ Cardiologist  □ Orthopedist □ Neurologist □ Psychiatrist  □ Dermatologist □ Others: _____________________  □ Other non-drug therapies: _________  □ No therapy known |
| Lack of concentration | □ Drug therapies  □ Medication as needed:________  □ Long-term medication:________  □ Rehabilitative measures  □ Physiotherapeutic measures  □ Problem-oriented conversations  □ Specialist referral  □ Internal specialist □ Pulmonologist □ Cardiologist  □ Orthopedist □ Neurologist □ Psychiatrist  □ Dermatologist □ Others: _____________________  □ Other non-drug therapies: _________  □ No therapy known |
| Cough | □ Drug therapies  □ Medication as needed:________  □ Long-term medication:________  □ Rehabilitative measures  □ Physiotherapeutic measures  □ Problem-oriented conversations  □ Specialist referral  □ Internal specialist □ Pulmonologist □ Cardiologist  □ Orthopedist □ Neurologist □ Psychiatrist  □ Dermatologist □ Others: _____________________  □ Other non-drug therapies: _________  □ No therapy known |
| Reduced mental health | □ Drug therapies  □ Medication as needed:________  □ Long-term medication:________  □ Rehabilitative measures  □ Physiotherapeutic measures  □ Problem-oriented conversations  □ Specialist referral  □ Internal specialist □ Pulmonologist □ Cardiologist  □ Orthopedist □ Neurologist □ Psychiatrist  □ Dermatologist □ Others: _____________________  □ Other non-drug therapies: _________  □ No therapy known |
| Chest pain | □ Drug therapies  □ Medication as needed:________  □ Long-term medication:________  □ Rehabilitative measures  □ Physiotherapeutic measures  □ Problem-oriented conversations  □ Specialist referral  □ Internal specialist □ Pulmonologist □ Cardiologist  □ Orthopedist □ Neurologist □ Psychiatrist  □ Dermatologist □ Others: _____________________  □ Other non-drug therapies: _________  □ No therapy known |
| Muscle or joint pain | □ Drug therapies  □ Medication as needed:________  □ Long-term medication:________  □ Rehabilitative measures  □ Physiotherapeutic measures  □ Problem-oriented conversations  □ Specialist referral  □ Internal specialist □ Pulmonologist □ Cardiologist  □ Orthopedist □ Neurologist □ Psychiatrist  □ Dermatologist □ Others: _____________________  □ Other non-drug therapies: _________  □ No therapy known |
| Headaches | □ Drug therapies  □ Medication as needed:________  □ Long-term medication:________  □ Rehabilitative measures  □ Physiotherapeutic measures  □ Problem-oriented conversations  □ Specialist referral  □ Internal specialist □ Pulmonologist □ Cardiologist  □ Orthopedist □ Neurologist □ Psychiatrist  □ Dermatologist □ Others: _____________________  □ Other non-drug therapies: _________  □ No therapy known |
| Metabolic dysfunction | □ Drug therapies  □ Medication as needed:________  □ Long-term medication:________  □ Rehabilitative measures  □ Physiotherapeutic measures  □ Problem-oriented conversations  □ Specialist referral  □ Internal specialist □ Pulmonologist □ Cardiologist  □ Orthopedist □ Neurologist □ Psychiatrist  □ Dermatologist □ Others: _____________________  □ Other non-drug therapies: _________  □ No therapy known |
| Thrombosis | □ Drug therapies  □ Medication as needed:________  □ Long-term medication:________  □ Rehabilitative measures  □ Physiotherapeutic measures  □ Problem-oriented conversations  □ Specialist referral  □ Internal specialist □ Pulmonologist □ Cardiologist  □ Orthopedist □ Neurologist □ Psychiatrist  □ Dermatologist □ Others: _____________________  □ Other non-drug therapies: _________  □ No therapy known |
| Loss of hair or skin disease | □ Drug therapies  □ Medication as needed:________  □ Long-term medication:________  □ Rehabilitative measures  □ Physiotherapeutic measures  □ Problem-oriented conversations  □ Specialist referral  □ Internal specialist □ Pulmonologist □ Cardiologist  □ Orthopedist □ Neurologist □ Psychiatrist  □ Dermatologist □ Others: _____________________  □ Other non-drug therapies: _________  □ No therapy known |
